# Insecticidal Activity of Plant Secondary Metabolites

**DOI:** 10.3390/plants11202804

**Published:** 2022-10-21

**Authors:** Giovanni Benelli, Filippo Maggi

**Affiliations:** 1 Department of Agriculture, Food and Environment, University of Pisa, Via del Borghetto 80, 856124 Pisa, Italy; 2 Chemistry Interdisciplinary Project (ChIP), School of Pharmacy, University of Camerino, Via Madonna delle Carceri, 62032 Camerino, Italy

Plant-insect interactions are one of the most fascinating fields of research attracting biologists, entomologists, botanists, as well as a wide range of multidisciplinary researchers. Focusing on plant-insect interactions can represent a useful approach to find new generation of green insecticides. Indeed, plant produce allelochemicals such as phenolic compounds, alkaloids and terpenoids targeting arthropods’ behavior and physiology. Thus, several compounds belonging to the above-mentioned chemical classes can represent ideal ingredients for developing sustainable Integrated Pest and Vector Management programs.

In this framework, the Special Issue “**Insecticidal Activity of Plant Secondary Metabolites**” is focused on recent advancements about the use of plant secondary metabolites against arthropod targets of health and agricultural importance.

The following topics have been covered by published papers:

- **Phytochemical analysis and biological evaluation of plant-borne secondary metabolites**. Badalementi et al. [1] performed a work on the isolation and structural elucidation of several bufadienolides from *Drimia pancration* and evaluated their acaricidal activity against a serious agricultural pest, the two spotted spider mite, *Tetranychus urticae*.
- **Lethal and sub-lethal effects of plant-borne insecticides and acaricides**. The Special issue attracted several significant studies on the topic. For example, Ebadollahi et al. [2] evaluated the efficacy of some essential oils from *Thymus* species against the stored product beetle *Rhyzopertha dominica*. Changbunjong et al. [3] investigated the fumigant and contact toxicity activities of bitter orange (*Citrus aurantium*) essential oil against the stable fly *Stomoxys calcitrans*. Plata-Rueda et al. [4] studied the sensitivity and behavioral response of the mealworm *Tenebrio molitor* towards oregano (*Origanum vulgare*) essential oil. Kostić et al. [5] tested three Apiaceae essential oils, namely anise (*Pimpinella anisum*), dill (*Anethum graveolens*) and fennel (*Foeniculum vulgare*) against *Lymantria dispar,* showing them as potential agents for gypsy moth control. Giordani et al. [6] and Wandjou et al. [7] outlined the promising toxicity of essential oils extracted from Chilean Patagonian and Cameroonian plant species against insects of agricultural (*Spodoptera littoralis*) and public health importance (*Culex quinquefasciatus* and *Musca domestica*).
- **Repellent effects of plant-borne secondary metabolites on hard ticks**. Alanazi et al. [8] studied the acaricidal and repellent activity of the cardamom (*Elettaria cardamomum*) essential oil against the tick *Hyalomma anatolicum*.
- **Modes of action and novel formulations of green insecticides**. Darrag et al. [9] proposed an efficient cell suspension technique to produce basil (*Ocimum basilicum*) extract containing secondary metabolites effective against the red palm weevil *Rhynchophorus ferrugineus* larvae and adults.

Finally, Spinozzi et al. [10] reviewed literature about *Acmella oleracea* highlighting its potential as insecticidal and acaricidal agent. Indeed, they provided the scientific basis for the industrial exploitation of this plant in the preparation of botanical insecticides and acaricides effective against several key arthropod pest and vector species.

Overall, we are grateful to all the co-authors for supporting this Special Issue and hope the published papers will represent the basis for the employ of natural products into green formulations to be exploited in real-world entomological applications.
